# Monitoring of gas station attendants exposure to benzene, toluene, xylene (BTX) using three-color chromosome painting

**DOI:** 10.1186/1755-8166-7-15

**Published:** 2014-02-27

**Authors:** Fábio Santiago, Gilda Alves, Ubirani Barros Otero, Marianne Medeiros Tabalipa, Luciano Rios Scherrer, Nadezda Kosyakova, Maria Helena Ornellas, Thomas Liehr

**Affiliations:** 1 Departamento de Patologia e Laboratórios, Faculdade de Ciências Médicas, Universidade do Estado do Rio de Janeiro, Rio de Janeiro, Brasil; 2 Programa de Pós-Graduação em Ciências Médicas, Faculdade de Ciências Médicas, Universidade do Estado do Rio de Janeiro, Rio de Janeiro, Brasil; 3 Laboratório de Genética Aplicada, Serviço de Hematologia, Instituto Nacional de Câncer, Rio de Janeiro, Brasil; 4 Unidade Técnica de Exposição Ocupacional, Ambiental e Câncer, Coordenação de Prevenção e Vigilância, Instituto Nacional de Câncer, Rio de Janeiro, Brasil; 5 Sociedade Brasileira de Oncologia Clínica, Belo Horizonte, Minas Gerais, Brasil; 6 Jena University Hospital, Institute of Human Genetics, Friedrich Schiller University, Kollegiengasse 10, D-07743 Jena, Germany; 7 Instituto Nacional de Câncer, Serviço de Hematologia, Laboratório de Genética Aplicada, Pç. da Cruz Vermelha, 23, 6° andar, 20230-130 Rio de Janeiro, RJ, Brazil

**Keywords:** Benzene, Toluene, Xylene, Monitoring, Cytogenetic, Painting, Chromosome

## Abstract

**Background:**

Chronic exposure of BTX (benzene, toluene, xylene) may lead to progressive degeneration of bone marrow, aplastic anemia and/or leukemia. In Brazil there is no self-service fuel in gas stations and attendants fill the fuel themselves. Due to this they are chronically exposed to high concentration of BTX. Occupational exposure to benzene has been associated with increased chromosomal aberrations in peripheral blood lymphocytes. Fluorescence in situ hybridization (FISH) using whole chromosome painting (wcp) probes allows the rapid detection of chromosomal aberration. In the present study three-color wcp probes for chromosomes 1, 2 and 4 were used for monitoring 60 gas station attendants.

**Results:**

Blood tests were done and interviews were conducted for each worker. For searching for possible associations between the clinical characteristics and the frequency of chromosomal aberrations the workers were divided into two groups (≤ 10 chromosomal abnormalities per 1,000 metaphases and > 10 chromosomal abnormalities per 1,000 metaphases).The studied workers had a low median age (36 year), albeit long period of BTX exposure (median was 16 years). Low prevalence of smoking and moderate consumption of alcoholic beverages were found in this population. The cytogenetic analysis showed 16.6% (10/60) of workers with a high frequency of chromosomal abnormalities (>10 chromosomal abnormalities per 1,000 metaphases). Translocations were the most frequently observed chromosome aberration. The statistical analysis revealed highly significant differences in skin color (p = 0.002) and a weak significant differences in gender (p = 0.052) distribution between the two groups.

**Conclusion:**

16.6% of the studied population showed elevated frequencies of chromosomal abnormalities, which is highly likely to be correlated with their exposure to BTX during their work. Therefore, further studies are needed for better characterize the work associated damage of the genome in gas station workers. It is necessary to better understand the risks that these workers are exposed, so that we can be effective in preventing diseases and maintaining the health of these workers and possibly the offspring.

## Background

In contrast to many developed countries, there are no self service fuel in gas stations in Brazil. Filling in fuel depends on attendants who are chronically exposed to BTX (benzene, toluene and xylene) during the work time. It is well known that benzene induces myelotoxicity in humans; the role of xylene and toluene is still unclear, thus, here we concentrate on the best studied substance among those three, benzene. It causes a variety of hematological disorders including aplastic anemia, myelodysplastic syndrome, and acute myelogeneous leukemia (AML) [1-4]. Benzene is metabolized in the liver to its primary metabolite phenol by cytochrome P4502E1 (CYP2E1) through the benzene oxide intermediate, and is subsequently metabolized by CYP2E1 to hydroquinone (HQ) [5,6]. HQ is transported to the bone marrow and oxidized to benzochinones, which eventually releases reactive oxygen species (ROS) damaging hematopoietic cells [5,7].

Many studies have been carried out to determine the hematological alterations and chromosomal aberrations (CA) in benzene exposed workers [8-11]. Reported genetic damages caused by benzene include sister chromatid exchange, DNA cross-linking, DNA adduct formation, and impairment of DNA repair mechanisms [12]. Other studies reported increased levels of chromosomal anomalies such as aneuploidies including monosomy of chromosomes 5 and 7 and trisomies of chromosomes 8 and 21, in the blood lymphocytes of apparently healthy Chinese workers exposed to high levels of benzene (median: 31 ppm, range: 1.6–328.5 ppm). It is well known that these kinds of alterations are also commonly found in leukemia and myelodysplastic syndrome [7,10,13]. Thus, chromosomal aberrations may be a precursor of future leukemia risk and other cancers. Additional studies have further strengthened the association between increased levels of CA in human lymphocytes and future cancer incidence and mortality [13,14].

Fluorescence in situ hybridization (FISH) using whole chromosome painting (wcp) libraries opened new insights in studying the CA in people exposed to mutagens and in delimiting individuals at risk [15]. This approach allows the rapid detection of translocations and other cytogenetic alterations, enabling new possibilities of cytogenetic dosimetry. This kind of test can also be used to address the chromosomal rearrangements detectable in individuals exposed to benzene and delimiting their individual cancer risk [1,15].

The aim of this study was to describe the cytogenetic changes on chromosomes 1, 2 and 4 in gas station attendants from Rio de Janeiro, Brazil, that had occupational exposition to BTX. The obtained results were used as an indicator for chromosomal damage as a whole, which happened in this population due to their work exposure.

## Results

### Health report

The gas station attendants routinely work for 6 days a week, during 8 hours or more per day. As it can be deduced from Table 1 the median time of employment in this activity was 16 years and their median age was 36 years. A low prevalence of smoking (15%) and a moderate consumption of alcoholic beverage (65%) were reported. No illicit drugs consumption (marijuana, cocaine and ecstasy) and no high consume of alcoholic beverages were described.

**Table 1:** Biometrics data (clinical and demographic) of gas station attendants

**Biometrics data**	**Results**	
**Gender**		
Men	50 (83.3%)	
Women	10 (16.7%)	
Age (years)	36 (± 13.5)	
Duration of exposure (years)	16 (±11.8)	
Smokers	9 (15%)	
Ex-smokers	9 (15%)	
Illicit drug consumption	0 (0%)	
Drinking	39 (65%)	
Ex-drinking	3 (5%)	
**Blood test**	**Men**	**Women**
Erythrocytes (million/μL)	4.9 (±0.32)	4.5 (±0.26)
Hemoglobin	14.4 (±1.14)	12.7 (±1.07)
Hematocrit (%)	42.3 (±2.84)	37.4 (±2.91)
Mean corpuscular volume (fL)	84.9 (±4.55)	84.9 (±4.26)
Leukocytes(/uL)	7025 (±1677.6)	
Neutrophils(%)	57 (±9.26)	
Typical lymphocytes	33 (±8.39)	
Basophils (%)	0.4 (±0.25)	
Eosinophils (%)	2.5 (±2.52)	
Monocytes (%)	7.1 (±1.76)	
Platelets (mil/μL)	230 (±54.05)	
Gamma-GT (U/L)	32 (±44.2)	

Gamma GT- Gamma glutamyl transpeptidase.

In the medical history of workers vision impairment (22.6%) was the complaint most frequently reported, followed by osteoarticular*^a^ (18.5%), cardiovascular (16.0%) and respiratory tract diseases (12.6%). Hematological diseases (1.8%) were scarce, and no neoplasic diseases were reported.

### Cytogenetic data

**Figure 1:**
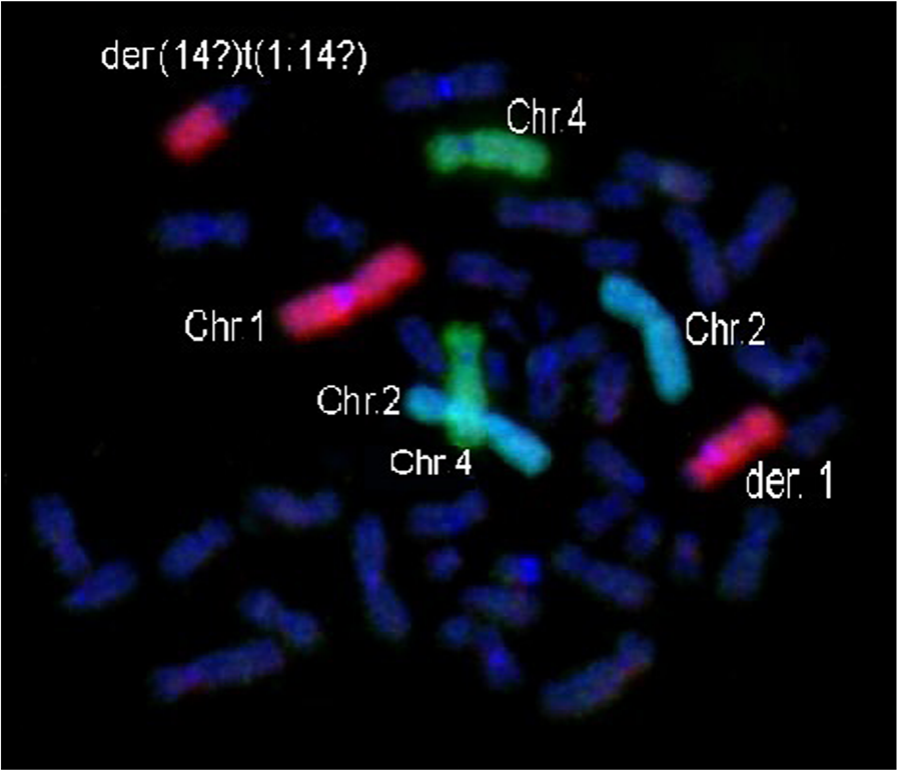
**The figure shows a translocation of chromosome 1.** The home made probes were conjugated with spectrum Orange for painting the chromosome 1 (red in the picture); spectrum FITC for painting the chromosome 2 (light blue in the picture) and spectrum DEAC for painting the chromosome 4 (green in the picture). DAPI was used with anti-fade solution (dark blue in the picture).

A high frequency of CA (> 10 chromosomal abnormalities per 1,000 metaphases) was found in 16.6% (10/60), whereas 83.4% (50/60) of workers showed no aberrations or less than 10 chromosomal abnormalities per 1,000 metaphases. Table 2 shows the CAs found on molecular cytogenetic analysis and Figure 1 shows an example of an abnormal metaphase.

**Table 2:** Chromosomal abnormalities of gas station workers

**Number**	**Chromosomal abnormalities**
1	chrb(1); del(1),del(2);chrb(1);t(2;?);t(2,?)
2	chrb(1);chrb(4)
3	t(1;2;?),t(2;?),t(4,?)
4	t(1;?),chrb(1); t(2;?)
5	ace(1)
6	−1,ace(1);del(4p);t(1;?)
7	t(2,?)
8	t(1;?), t(2;?);del(1),del(4)
9	t(1;2)
10	t(1;?)
11	der(1), t (1;2)
12	del(2);t(1;?);t(4;?);ace(4)
13	+4
14	del(2)
15	del(4)
16	del(2)
17	del(1), del(2); -4
18	−4,ace(4)
19	t(2;?)
20	−4
21	chrb(4)
22	t(1;?)
23	del(4)
24	−1
25	chrb(2)
26	−1
27	t(4;?)
28	t(1;?)
29	t(2;?)

A compared to normal controls (see Discussion) elevated frequency of CAs (9.3 per 1,000 metaphases analyzed) was found in the gas station workers population. Among the total CAs the translocations were most frequently found (43.6%), followed by deletions (23.7%); monosomies (10.9%); chromosomal breaks (12.7%); chromosomal fragments (7.3%) and trisomies (1.8%).

Translocations of chromosome 2 were responsible for 20% of total CAs, followed by translocation involving chromosome 1 (18.2%) and gross deletions within chromosome 2 (9.1%). Chromosome 1 showed the highest percentage of CAs (41.8%) that corresponded to: translocations (18.2%), chromosome breakage (7.2%), deletions (7.2%), monosomies (5.4%), and formation of chromosome fragments (3.6%). Chromosome 4 had the lowest percentage of CAs observed (27.3%). Among the CAs of chromosome 4 the most common were deletions (7.2%), followed by translocations (5.4%), monosomies (5.4%), chromosome breaks (3.6%), chromosome fragments (3.6%) and trisomies (1.8%).

The number of translocations was compared to other chromosomal aberrations that were found. Table 3 shows that significantly differences were present, pointing that monosomies (p = 0.022), trisomies (p = 0.00) and chromosomal fragments (p = 0.007) were less frequent than translocations in chromosome 1. In Table 4 the same analysis was done for chromosome 1 and 2. The translocation of chromossome 2 was the most frequent aberration seen in this chromosome. The overall-involvement of translocations in rearrangements did not show any significant differences for the number of translocations of chromosome 4 compared to the other chromosomal aberrations.

**Table 3:** Statistical analysis between translocations and others chromosomal abnormalities of chromosomes 1, 2 and 4

	**Number of chromosomal aberrations**	**P-valor**
Translocation	24	Aberration compared
Chromosome breakage	7	0.000
Chromosome fragment	4	0.000
Deletion	13	0.026
Monosomy	6	0.000
Trisomy	1	0.000

**Table 4:** Statistical analysis between translocations and others chromosomal abnormalities of chromosome 1 and 2

	**Number of chromosomal aberrations**	**P-valor**
**Chromosome 1**		
Translocation	10	Aberration compared
Chromosome breakage	4	0.055
Chromosome fragment	2	0.007
Deletion	4	0.055
Monosomy	3	0.022
Trisomy	0	0.000
**Chromosome 2**		
Translocation	11	Aberration compared
Chromosome breakage	1	0.000
Chromosome fragment	0	0.000
Deletion	5	0.039
Monosomy	0	0.000
Trisomy	0	0.000

### Association between the cytogenetic results and clinical and demographic data

For possible associations between the clinical characteristics and the frequency of CAs the subjects were divided into two groups (≤ 10 CAs per 1,000 metaphases = group 1, and > 10 CAs per 1,000 metaphases = group 2). The statistical analysis revealed highly significant differences in skin color distribution between the groups (p = 0.002). Similarly, we found, when statistical analysis were done, differences only concerning blacks and whites between the two groups (p < 0.01, OR = 9.02 and 95% CI [1.54 to 97.77]). It was observed that blacks have a lower number of CA than whites. Also we observed on the gender analyze that 88% of male workers were in group 1 and 12% in group 2, while it were 60% of female workers in the group 1 and 40% in group 2.The statistical analysis revealed weak significant differences in gender distribution between the two groups (p = 0.052, OR = 4.71 and 95% CI [0.76 to 28.05]).

To assess whether the frequency of CAs has association on hematological and biochemical parameters Table 5 was established. No significant associations were found between frequency of chromosomal aberration and whole blood cell count (WBC), red blood cell count (RBC), Gamma-glutamyltransferase (p > 0.05) and platelets count (p = 0.059), however for the latter p-value was borderline.

**Table 5:** Associations between the frequency of chromosomal abnormalities and biometrics (clinical and demographic) data

**Biometrics data**	**≤ 10 chromosomal abnormalities per 1000 metaphases**	**>10 chromosomal abnormalities per 1000 metaphases**	**p-value**
Gender			0.052
Women	6	4	
Men	44	6	
Age (year)	34.00 (±13.73)	42.00 (±12.51)	0.177
Time of employment (year)	12.50 (±12.53)	18.00 (±8.41)	0.433
Skin color			0.002
Black	33	2	
White	14	8	
Native American	2	___	
Asian	1	___	
Platelets (mil/μL)	231.50 (±52.41)	195.0 (±61.82)	0.059
Gamma-GT (U/L)	32.0 (±48.47)	32.5 (±12.58)	0.944
Leukocytes (/μL	7020 (±172)	6930 (±151)	0.835
Neutrophils (%)	56.58 (±9.66)	57.00 (±5.48)	1.000
Eosinophils (%)	2.80 (±2.65)	1.84 (±1.49)	0.312
Basophils (%)	0.35 (±0.25)	0.27 (±0.27)	0.585
Typical lymphocytes (%)	33.20 (±8.94)	31.20 (±4.89)	0.565
Monocytes (%)	7.10 (±1.84)	7.28 (±1.43)	0.866
**Women**			
Erythrocytes (million/μL)	4.48 (±0.22)	4.51 (±0.35)	0.521
Hemoglobin	13.15 (±0.9)	12.30 (±1.44)	0.669
Hematocrit (%)	38.80 (±3.01)	36.00 (±3.11)	0.915
Mean corpuscular volume (fL)	84.45 (±4.17)	84.90 (±4.88)	0.915
**Men**			
Erythrocytes (million/μL)	4.92 (±0.48)	5.00 (±0.13)	0.855
Hemoglobin	14.30 (±1.21)	14.70 (±0.71)	1.000
Hematocrit (%)	42.90 (±3.06)	41.80 (±1.37)	0.944
Mean corpuscular volume (fL)	84.90 (±4.89)	84.90 (±2.42)	0.944

Gamma-GT - Gamma-glutamyltransferase. Gender: OR (95% CI) = 4.71 (0.76 - 28.05). Skin color: Native American and Asian were excluded from statistical analysis because the sample size were below 5; OR (95%) = 9.02 (1.54 a 97.77).

## Discussion

In the present study, the frequency of CAs in peripheral blood lymphocytes of Brazilian gas station workers was used as an effect biomarker of BTX exposure. The chromosomes pairs 1, 2 and 4 represent together 21.87% of human genome. Thus they were chosen for the cytogenetic paint analysis as representatives of the entire human genome as previously described by Verdorfer and colleagues [15].

A questionnaire analysis showed a population with a low median age, albeit with long period of exposure. We did not detect inappropriate health behaviors on life style questions. The self-reported medical history showed that the main health problems were related to acute symptoms; reported sight changes appeared to be mainly associated with direct irritant action of fuel vapor in eyes (no masks are used). Osteoarticular diseases were most likely due to the long period of time in physical labor. Hematological and neoplasic diseases were rarely/not reported by workers, which was most likely due to the quiet character of the natural history of these diseases and the fact that they lead quickly to absence from work. Also they are late manifesting diseases, which might not be present in the on average young population studied.

It should be remembered that fuel volatile fraction contains chemicals other than BTX that may act as aneugens or clastogens. It is likely that the workers in this study were exposed simultaneously to several other complex chemicals. However, considering the different behaviors of environment chemicals once released, the significant relationship observed with respect to BTX concentration, indicates a specific role for BTX, mainly for the benzene, in this association [9,16].

Several studies examined cytogenetic endpoints in subjects exposed to petroleum fuels, auto exhaust, or other organic solvents [1,10,17-23]. The benzene exposure has been strongly associated with increased chromosomal abnormalities in the lymphocytes in individuals without diseases [22]. Verdorfer and colleagues analyzed with same technique different groups of individuals with different types of exposures [15]. In our study the workers had a higher frequency of chromosomal abnormalities when compared with Verdorfer groups (control group, military occupied in nuclear area and radiology workers) [15].

In the present search were found 10/60 of workers with high number of chromosome abnormalities (all workers with ≥20 abnormalities per 1,000 metaphase). It is worth to note that in a normal control group only 4/60 individuals with high number of chromosome abnormalities could be expected (≥20 abnormalities per 1,000 metaphase) [15].

CAs have a direct association with malignancy. An induced chromosomal instability could also predispose cells to further mutations and by that to an increased risk of malignant transformation [24]. Several researchers studying acute exposure of workers to fuel or organic solvents reported gaps and chromosomal breaks as the chromosomal abnormalities most often detected [16]. While chromosomal translocations were described as markers of chronic exposure that dating back up several years of benzene exposure, so the number of translocations may be a parameter for long term exposure to benzene or BTX [15,25,26]. Thus, the elevated involvement of chromosomal translocations found in this study should be most likely due to the long years of workers exposure. Also, chromosome breaks detected in conventional cytogenetic studies cannot be detected in FISH (own unpublished data).

It is worth remembering that significant decreases of WBC, RBC and platelet counts already been observed in human populations exposed to high levels of benzene [11,27]. However, we did not find a relationship between the frequency of CAs and the rates of WBC or RBC counts in the studied population. The presence of isolated thrombocytopenia is a change which was previously described in literature [28]. Remarkably, the statistical positivity association of platelet decreased counting with frequency of CAs was closer. Further and longer studies are needed to associate the effects of BTX exposure between frequency of CAs and hematological changes with broad range of exposures.

In our study a weak association between gender and frequency of CAs were found. It is serious concern the possibility of women’s genome be severely more affected by BTX exposure. Several epidemiological studies support the idea that genotoxic and nongenotoxic events following benzene exposure may be initiators of childhood leukemia in utero [1]. Another study on AML have shown that the disease is usually initiated in utero because the leukemic translocations and other genetic changes are present in blood spots collected at birth [29-31]. Thus, mother exposure to benzene could be just as important as childhood exposures in producing childhood AML and acute lymphoblastic leukemia.

## Conclusion

The number of workers with high amount (10/60) and the high frequency of CAs (9.2 per 1,000 metaphase) found shows how necessary it would be to expand this study nationwide, since Brazil has great ethnic and cultural diversity. The results obtained are valuable, but were only obtained from 5 gas stations in Rio de Janeiro city, a pilot study. It is necessary to better understand the risks that these workers are exposed, so that we can be effective in preventing diseases and maintaining the health of these workers and possibly the offspring.

## Methods

This study was approved by the local ethics committee (Instituto Nacional de Câncer – INCA, Brazil). All subjects were informed for each individual about the nature of the study, the potential benefits, and the risks. Participation was voluntary and written informed consent was obtained from each subject before study participation.

### Population study

The study included 50 male and 10 female workers recruited on 6 gas stations, in Rio de Janeiro city. A trained interviewer questioned the members of the study population regarding their age, sex, race, life-style (smoking habits, alcohol and illicit drugs consumption, etc.) and medical and work histories.

Peripheral blood samples were collected for complete hemogram, biochemistry tests and cytogenetics. The cytogenetic analyses were made for delimiting workers at risk and for allowing associations between the frequency of CA and clinical characteristics.

### Chromosome preparation

Blood samples, 2 ml of heparinized whole blood, were collected by venipuncture. For each sample two cultures were performed according standard technique of lymphocyte cultures. Chromosomes were prepared according to standard procedures after 48 hours of cultivation [32].

FISH was done as previously reported [15] using home made wcp probes for chromosomes 1, 2 and 4 [33]. Per gas station worker 100 metaphases should be analyzed; this was possible in 47 cases. In the remainder 13 cases 34 to 97 metaphases were available.

### Hematological and biochemistry analysis

The hematological analysis consisted of complete hemogram measuring of hemoglobin, hematocrit, mean corpuscular volume and white blood cell counts. The biochemistry analysis consisted of measuring gamma glutamyl transpeptidase (Gamma GT), aspartate transaminase (AST), glutamic pyruvic transaminase (TGP), lactate dehydrogenase (LDH), bilirubin, creatinine and c-reactive protein.

All blood tests were analyzed in the central laboratory of INCA, according to standard haematological methods.

### Statistical analysis

Subjects were divided into two groups (≤ 10 chromosomal abnormalities per 1,000 metaphases and > 10 chromosomal abnormalities per 1000 metaphases) and compared to clinical characteristics (age, time of employment, race, hemoglobin, leukocytes, etc.) by either *Mann–Whitney* test or *chi-square* test (if either quantitative or dichotomic variable). The *Mann–Whitney* test was performed because the variables quantitative did not have a Gaussian distribution in most of situation. For cytogenetic analyses the Chi-Square Goodness-of-Fit test was performed to evaluate statistical differences of chromosomal aberrations distribution among chromosomes 1, 2 and 4. For all statistical tests p < 0.05 was considered significant. All analyses were carried out using the PASW software (Version 18, Inc., Chicago, IL, USA).

## Endnotes

^a^*Osteoarticular is defined as disease relating to, involving, or affecting bones and joints.

## Competing interests

The authors declare that they have no competing interests.

## Authors’ contributions

GA, UBO and MHO designed the study and applied for Research Ethics Board approval. MMT and FS recruited the workers. The cytogenetic data were analyzed by NK and FS. LRS analyzed all data. FS prepared the manuscript draft with important intellectual input from TL, GA and MH. All authors approved the final manuscript and had complete access to the study data.
